# Transforming Psychiatry into Data-Driven Medicine with Digital Measurement Tools

**DOI:** 10.1038/s41746-018-0046-0

**Published:** 2018-08-22

**Authors:** Honor Hsin, Menachem Fromer, Bret Peterson, Collin Walter, Mathias Fleck, Andrew Campbell, Paul Varghese, Robert Califf

**Affiliations:** 1 Verily Life Sciences, 269 East Grand Avenue, South San Francisco, CA 94080 USA; 2 Verily Life Sciences, 355 Main Street, Cambridge, MA 02142 USA; 3 Department of Computer Science, Dartmouth College, Hanover, NH 03755-3510 USA; 4 Duke Forge, Duke University, Durham, NC 27708 USA; 5 Department of Medicine, Stanford University, Stanford, CA 94305 USA; 6 Verily Life Sciences, South San Francisco, CA 94080 USA

**Keywords:** Predictive markers, Psychiatric disorders

## Abstract

Psychiatry has been limited by historically rooted practices centered primarily on subjective observation. Fields such as oncology have progressed toward data-driven clinical decision-making that combines subjective clinical assessment of symptoms and preferences with biological measures such as genetics, biomarkers, imaging, and integrative physiology to derive quantitative risk scores and decision support. In contrast, psychiatry has just begun to scratch the surface of measurement-based care with validated clinical questionnaires. An opportunity exists to improve modern psychiatric care with novel data streams from digital sensors combined with clinical observation and subjective self-report. The prospect of integrating this complex information with modern computational and analytical methods could advance the field, both in research and clinical practice. Here we discuss this possibility and propose some key priorities to enable these innovations toward improving clinical outcomes in the future.

*“Let no one deceive himself: trying to understand another human being’s emotional life is fraught with potential error … As intuition is greatly influenced by one’s own prejudices and needs, it lends an air of deceptive yet powerful plausibility. This is especially worrying as we have no objective yardstick for this confidence.”*

– Emil Kraepelin, The Manifestations of Insanity, 1920^1^

Despite modern advances in the scientific understanding of psychiatric disorders, clinical practice today remains bound to intuition-based assessments that have persisted since Kraepelin’s era. For the most part, psychiatric practice continues to rely upon heuristic-based decisions that are frequently reinforced without good comparative evidence, and often for the sake of maintaining a therapeutic relationship for lack of a better alternative.^2^ At best, this approach allows practice to remain patient-centered, but at worst, this approach could be maintaining biases that are preventing patients from receiving optimal care.^3^ Selecting medications to treat depression based on potential advantage of an anticipated side effect (such as sedation) is an example of such clinical practice–therapeutic adherence may be improved, but how will we know we are not withholding better treatments from patients as a consequence of our own biases? Here, we propose that today’s era of technological innovations in wearables and mobile devices offers a unique opportunity to redefine these limits of practice toward a new, data-driven future.^4,5^

Specifically, measurement tools that integrate information over time, such as smartphone-based monitoring of circadian rhythm, physical activity, and social trends, may allow us to quantify behavior at levels of granularity never before obtained over long periods of time and at scale. Supplemented with self-reported outcomes, this approach to measurement could enable testing of clinical heuristics already in use, and create an opportunity for a data-scientific approach toward understanding the clinical significance of behavioral phenomena already encountered in daily practice. Furthermore, these tools may enable personalized care in specific clinical situations where measured constructs and interventions are appropriately matched. Activating these insights and adding a transformational data-driven dimension to the field could have a major impact on clinical care.

## Assumptions about Digital Measurement

Enthusiasm for the positive impact of “digital phenotyping” is built on fundamental assumptions.^5,6^ One is that continuous measurements will offer useful signals over and beyond sporadic measurements obtained at clinic visits. Similar to all of medicine, limitations in recording, storing, and computing have relegated research and practice to periodic assessment influenced heavily by recall of what happened between visits. Rather than recalling the clinical course between physical visits, we can now attempt to measure them. Evidence exists in other branches of medicine that continuous measurement in ambulatory settings (i.e., the “real world”) may be better at predicting clinical outcomes than in-person visit measurements alone. For example, ambulatory 24 h systolic blood-pressure measurements are more strongly associated with cardiovascular and all-cause mortality than in-clinic systolic pressure.^7^ Interventions may also be targeted for clinical impact, just as implantable cardioverter defibrillators that continuously monitor cardiac rhythm in the real world can deliver automated shocks to reverse sudden cardiac death.

Continuous objective data collected in mental health populations are already demonstrating how these measurements could augment conventional periodic, subjective assessments. For example, one study showed that sleep actigraphy measures modestly outperformed a more conventional depression symptom assessment (Beck Depression Inventory) at predicting future suicidal ideation in a cohort of 50 young adults.^8^ Features of heart rate variability obtained in defined continuous segments appear to be predictive of a posttraumatic stress disorder diagnosis with an area under the receiver operating curve (AUC) of 0.86 (23 diagnosed subjects and 25 control subjects).^9^ Emerging work with smartphone sensors raises the possibility that continuous human–computer interaction metrics may predict neuropsychological function in healthy individuals (as assessed by gold-standard psychometrics in 27 subjects),^10^ and even delays in on-device survey completion rates appear to be associated with subsequent self-report of negative symptoms in patients with schizophrenia (16 patients).^11^ These findings highlight that continuous measurements are showing early signs of promise at providing additional value beyond traditional subjective reports.

A second assumption is that integrative analysis of data from measurement tools, combined with clinical measures and assessment of outcomes, may uncover behavior-symptom-environment clusters that could inform new subtypes of disease that were previously unknown. This dimension could be akin to the transformation of oncology into treatment based on tumor genetics rather than organ of origin. A fundamental reclassification in cancer was not possible until the genome could be sequenced and tumor sequences could be matched with clinical outcomes and response to therapy. Striking examples of this impact, such as the United States Food and Drug Administration’s recent approval of pembrolizumab for the first genomic signature (anatomic site-agnostic) indication,^12^ continues to inspire attempts at real-world evidence collection of clinical and genomic cancer data.^13^

Similarly, behavioral and physiological measurements could define axes of clinical significance, which, when taken together, map onto different prognostic outcomes or indicated treatments. This interesting possibility in mental health has been suggested by recent studies applying machine learning methods to functional magnetic resonance imaging (fMRI) data of brain connectivity patterns obtained from depressed individuals,^14^ as well as to self-report symptom data obtained from large clinical trial datasets of depression treatment.^15,16^ Here, analyses using data science approaches posited the existence of clinical depression subtypes with implications for differential treatment response profiles: neural connectivity biomarkers significantly outperformed clinical symptoms alone at prediction of response to transcranial magnetic stimulation therapy (in 154 depressed subjects)^14^ and selected baseline self-report profiles significantly outperformed chance at predicting antidepressant treatment response for symptom clusters (across 4039 patients from the Sequenced Treatment Alternatives to Relieve Depression trial).^16^ Digital measurement tools may similarly refine traditional boundaries of psychiatric diagnosis by potentially stratifying patient characteristics in a way that is clinically actionable.

## Toward Data-driven Practice

What are some steps that can be taken in this new world of digital phenotyping to improve clinical outcomes? We propose four areas that could help transform this early vision into meaningful impact: (1) collect data from real world individuals and conditions beyond the current constraints of clinical practice; (2) invest in data science and analytics, and the interface of quantitative methods with clinical care; (3) uphold the patient experience within appropriate bounds of self-education and self-empowerment; and (4) work with clinicians to develop effective approaches to incorporate the use of data in daily practice.

### Start from the real world

Although all fields of medicine have struggled with the portability of findings from clinical trials into the real world, psychiatry has been particularly challenged. Patients enrolled in most research studies have been carefully selected and frequently screened out for comorbidities in order to optimize response and follow-up.^17^ Although for some types of questions, rarified populations are needed to evaluate a mechanistic hypothesis, these trends have understandably led to legitimate skepticism about the generalizability of many clinical trials.^18^ There is a need to conduct trials with research participants representing patients seen in current ambulatory contexts so that findings can be translated better into practice.^19,20^

Wearable and smartphone devices, in contrast to genetic tests, analyte sampling, and neuroimaging, are immediately accessible and affordable to patients and researchers today.^5^ A potential synergy thus emerges from the ubiquitous availability of behavioral sensor technology and resulting data, juxtaposed with the need for real-world evidence generation, spanning the spectrum from observational cohort studies to randomized treatment or implementation trials. Digital phenotyping has the potential to become clinically useful precisely because it can be used to measure data ecologically from living populations, yet also allows a focus on a specific outcome or actionable decision in order to guide future clinical practice.

### Invest in data science and analytics

With streams of continuous data, investments in appropriate data science and analytics are certainly required. Measurement tools in particular often carry a temptation to confuse clinical-grade and consumer-grade analytics. As studies have shown, consumer-grade evidence may not be reliable enough to serve as the basis for algorithms to inform medical decisions, and few clinical trials have been done to validate the potential benefits for clinical populations or use cases.^21^ Investments in scrupulous data collection, secure data management, quality control of data streams, carefully validated feature engineering of the collected data, and rigorous predictive and causal modeling^22^ for clinical use are prerequisites for digital phenotyping to have meaningful impact in clinical care.

The potential for data science to drive improved interpretation of clinical data holds great promise to energize the field. Machine learning algorithms in Chekroud et al.^16^, for example, suggested a focused survey of baseline interview questions that could aid prediction of remission with specific antidepressants. Using large-scale datasets, robust evidence-based modeling approaches for actionable prediction and phenotyping of outcome could bypass time-consuming trial-and-error approaches of testing particular heuristics. As an example from the study of psychiatric genetics, scaled genome-wide association studies have disproven the previous hypothesis of a large contribution of rare variants of large effect size.^23^ Analogously, learning whether or not decreased physical activity, for example, is of large enough clinical significance to inform treatment change (and if so, for which patients) could save the field from another decade of the “deceptive yet powerful plausibility” Kraepelin warned against.

### Uphold the patient experience

Parsimony of data collection (limiting to that which is needed), transparency to users along with control of privacy settings, and protection of data should be cornerstone values for any measurement tool that hopes to earn patients’ trust, adoption, and continued use.^24^ Specifically, data collection and management will need to be performed in a secure, confidential, and quality-controlled manner, in compliance with clinical data protections. Transparency into how digital data are de-identified, stored, and accessed, including by potential third parties, is crucial. For lasting trust to develop, users need to be empowered to “opt-in” to data collection efforts with as clear control over their data as possible.

Recent trends in wellness apps and devices have primed users to these channels being used for health data collection, but these modalities also carry user expectations around receiving accordant value in exchange for the investment of effort and data. Such value may come from returning the data collected in a digestible format, suggesting specific actions based on the data, or surfacing relevant educational content and support resources within a compelling user experience. Developing such user-oriented features may improve adherence with measurement tools and even empower users to assume a greater sense of agency for personal health within reasonable bounds. However, it is important to maintain transparency with users regarding the ethical limits of returning health-related data, and to set appropriate expectations around the value of clinical information that is being measured and returned.

### Learn with clinicians

Similar to how machine learning of clinical imaging data has begun to redefine traditional roles of radiologists and pathologists,^25^ an influx of novel data and analytics would ultimately lead to a redefinition of the role of clinician teams who will need to interpret these data in the midst of the complex subjective issues in mental health care. The availability of these tools will necessitate clinician education to evaluate the clinical utility and validity of outputs derived from algorithms: clinicians will not need a detailed understanding of computational methods per se, but rather a familiarity with statistical measures of algorithm performance (e.g., AUC) to be able to interpret and apply clinical research to use cases as easily as a number-needed-to-treat result from a clinical trial. We see this evolution akin to how advances in precision medicine have motivated medical institutions to incorporate more integrated exposure of genetics into clinical training, in anticipation of widespread genome sequencing and genetic risk assessments becoming part of clinical care.^26,27^

With respect to specific clinical practices, simple guidelines may be considered in the future as well. An example is defining the use cases where “N of 1”^28^ digital phenotyping data and clinical judgment can meaningfully coexist without jeopardizing standard of care, as in the optimization of behavioral outcomes (e.g., alcohol consumption behavior). In comparison with clinical endpoints based on disorder categories (e.g., remission of major depressive episode), clinical behaviors are immediate, quantifiable, and modifiable. Thus, the greater proximity of measurement inputs to intervention outputs for health-related behaviors may allow for data-driven heuristics to play a greater role under appropriate supervision of clinical care. Additional uses may include careful monitoring during medication titration in specific cases (e.g., low iatrogenic addiction risk), relapse detection, patient self-regulation, and delivery of selected digital interventions.^29–33^ Engagement of ethical clinicians in defining the appropriate boundaries of personalized care using digital measurement tools is crucial to realize potential clinical benefits while protecting against potential harms.

## Conclusion

We believe that data-driven psychiatry is possible, and that digital measurement tools and analytics, as “objective yardsticks,” can help catalyze this future. With appropriate attention to real world clinical outcomes, data science, patient experience, and the role of clinical judgment with respect to standard of care, psychiatric practice can leapfrog into a modern era already occupied by other medical fields. For the sake of future patients, there is no better time for this investment than today.
